# Dying To Be Thin: Attachment to Death in Anorexia Nervosa

**DOI:** 10.1100/tsw.2005.95

**Published:** 2005-09-29

**Authors:** Yael Latzer, Zipora Hochdorf

**Affiliations:** ^1^ Eating Disorders Clinic, Division of Psychiatry, Rambam Medical Center, Haifa, Israel; ^2^ School of Social Work, Faculty of Social Welfare and Health Studies, University of Haifa, Haifa, Israel; ^3^ Faculty of Education, University of Haifa, Haifa, Israel; ^4^ Western Galilee College, Acco, Israel

**Keywords:** attachment styles, eating disorders, life, death, Anorexia Nervosa, human development, suicide, public health, Israel

## Abstract

Anorexia Nervosa (AN) usually follows a prolonged course accompanied by significant morbidity and high mortality. AN patients have been found to have elevated and attempted suicide rates, with suicide being the second most common cause of death in AN after the complications of the disorder itself. The suicide risk in AN is similar to that in major depression or conduct disorder and linked mainly to longer duration of illness, lower weight, bingeing and purging, impulsivity-related manifestations, comorbid substance abuse, and affective disorder. This paper reviews suicidal tendency and disturbed body image, death and eating disorders, and attachment and death with clinical implications related to AN.

## INTRODUCTION

Anorexia Nervosa (AN) usually follows a prolonged course accompanied by significant morbidity and high mortality[1]. According to the DSM-IV[2], one of the diagnostic characteristics of AN is denial. These patients tend to view their low weight as an accomplishment rather than as an affliction and, consequently, have little motivation for change. Their drive for thinness is considered egosyntonic[3].

As Yalom[4] described, the inner psychological state and struggle of AN patients with life and death seem to be characterized by “straddling the fence between living and not living.” Thus, one should raise the question as to what kind of dialog AN patients really have with life and death. What is “death’s meaning for life” for them[4]? Do they really wish to die? Are they attracted to death or are they just rejecting life? Is it a latent version of major depression? Is it an unconscious way to hurt one’s parents and significant others for not being attuned to one’s needs? Is there a relationship between body disturbances and suicidal tendencies? Is it a latent way to commit suicide? Is the wish to die the only way to have meaningful life?

## SUICIDAL BEHAVIOUR AND DISTURBED BODY IMAGE IN AN

AN patients have been found to have elevated and attempted suicide rates[5,6], with suicide being the second most common cause of death in AN after the disorder's complications itself[7]. The suicide risk in AN is similar to that in major depression or conduct disorder[5,7]. Suicidal tendency in AN is linked mainly with longer duration of illness, lower weight, bingeing and purging behaviour, affective disorder, impulsivity-related manifestations and comorbid substance abuse[8,9]. In addition, psychological characteristics associated with a greater rate of attempted suicide in AN include lower self esteem, perfectionism[7] and obsessive tendencies[8].

Researchers have demonstrated the important role of negative attitudes towards the body in increasing the tendency to self-destruction[10,11]. There is indication that the relationship between body image disturbances and suicidal tendencies in AN stems from the incorrect interpretation of interceptive stimuli, insensitivity to bodily functions, and lack of bodily control, causing distorted perception and detachment from the body. These may ultimately lead to self-neglect and self-destructive behavior, including attempts to commit suicide upon weight gain[12]. The lack of control over the body may be accompanied by a loss of any sense of pleasure[13]. Indeed, disturbances in body image, and particularly highly negative attitudes towards the body, may turn the basic fear of life of AN patients into self-destructive tendencies that not only reflect a wish to destroy the body, but also to regain a new sense of pleasure by controlling their whole existence, both emotional and physical[12].

## DEATH IDEATION AND EATING DISORDERS

The literature regarding death in the etiology of eating disorders is very scant, despite a possible relationship between eating disorders and suicidal tendencies or repulsion by life. In addition, various researchers[14,15] have found that the disturbed preoccupation with death plays a role in the development and maintenance of these disorders, especially in AN and perhaps in Bulimia Nervosa as well. Other researchers[16,17] employ Lifton's[18] concept of "Survivor's Guilt" to describe the behavior of the anorexic patient. This syndrome involves the onset of the disorder after the death or serious illness of a close relative, thus allowing the anorexic patient to behave as if she has become the dead relative[17].

Warren et al.[15], however, did not find differences between eating disorder (ED) patients and other clinical groups on variables such as fear, acceptance, or contemplation of death. In line with construct psychology[19], Warren[20] raised a counter-argument to the idea of death as a universal factor present in every ED patient. He suggested that the particular significance of death ideation must be determined on a case-by-case basis, taking into account the unique meaning for each patient. Accordingly, he argued that the theme of death would be highly salient in some cases and less so in others.

In order to clarify the perception of life and death among ED patients, Bachar et al.[21] used the differentiation between attraction to and repulsion by life and attraction to and repulsion by death as independent factors. They used the scale developed by Orbach et al.[22] to investigate ED patients' attraction to and repulsion by life and death[10,22,23,24]. The results indicated that ED patients were less attracted to and more repulsed by life than normal controls. No significant differences were found in death variables, either in attraction to or repulsion by death.

These results highlight the theoretical concept of Becker[25], who contended that the normal individual blocks the acknowledgment of the reality of death more effectively than the anorexic patient, who lacks this defense and thus allows the true nature of reality to appear. To some degree, a healthy preoccupation with death can give meaning and purpose to life. It is only the unhealthy denial of death that promises freedom from anxiety about it. Due to the strength of defenses that maintain the broken connection between life and death, most people do not consciously think about death[16].

Stein et al.[26] examined the relationship between body image and suicidal tendencies among AN patients as compared to hospitalized psychiatric patients (both suicidal and nonsuicidal). They found that AN patients and suicidal patients showed less attraction to but more repulsion by life, and more attraction to but less repulsion by death than the other two groups. AN patients and suicidal patients were different from the control groups insofar as they exhibited more negative attitudes towards their body, lower sensitivity to body signs, and elevated levels of depression and anxiety. Similar results were found by Orbach et al.[27] examining body image and attraction to life and death among inpatient psychiatric adolescent groups. The findings showed that the suicidal group differed from the two nonsuicidal groups in feelings towards body protection and body dissociation. However, Stein et al.[26] emphasized that AN patients had a higher body distortion experience.

## DRIVE FOR DEATH IN A THEORETICAL PERSPECTIVE

Many therapists and theoreticians have tried to understand the inter- and intrapersonal (familial) dynamics of patients suffering from AN. One of the first to attempt a description of the treatment of a 13-year-old AN girl was Frances Tustin[28]. The majority of her work focused on the understanding of autism and its implications in the treatment of neuroses. According to her theoretical concept, the AN patient keeps herself in a shell in order to avoid painful feelings. This autistic-type defense against pain stems primarily from the intensity of the fear of death and disintegration. Tustin's ideas stimulated and inspired other therapists, who developed therapeutic understandings that continue Tustin's work[29].

Some authors have explored the dialog of AN with life and death by referring to the denial of AN patients from a psychodynamic perspective. They claim that these patients play with the idea of death like a child in a game, pretending that they can disappear through death and return in a mystical way[16,30]. According to Bruch[31], AN patients feel guilty for giving in to the gross and vulgar demands of the body. Thus, they choose to live as the self, but to die as the body[32].

Other authors relate to the dialog of AN with life and death from an interpersonal theory perspective[12,31,33]. According to this viewpoint, AN patients are not attracted to death so much as they are seeking control over their life and a sense of identity. The symptoms represent a latent suicidal act as the result of feeling depressed for not achieving such control and thereby serve as a way to achieve false control.

An unconscious fantasy in the anorexic patient's starvation is a vision of impending death and becoming a walking corpse. This may happen in response to a parent identifying her as a future anorexic in order to replace an ambivalently loved relative who died[13,34,35]. The fear of the parent's unconscious death wishes now directed at the child through overprotectiveness. The parent acts as if death is imminent, and the anorexic child's appearance becomes the cruel parody of the parent's most horrible fears[13,35].

Goodsitt[13] described the theme whereby a patient would have a fantasy of her parents grieving over her dead body. She could then gloat that her parents finally realized the wrong they had done her, but that now it was too late. In one case, the patient kept her bedroom darkened, except for candlelight, and created an altar in front of which she prayed and fantasized that she was her parents' human sacrifice. This dark sacrificial room, permeated with the sense of death, was meant to deliver a cruel blame to her parents that "you have killed me". She reveled that when her mother hugged her, she would be horrified in feeling only bones. She wanted to look sick, ugly, and disgraceful, and that her appearance would evoke shock in others.

Lifton[18] described the AN patient as an individual who was too afraid to live fully, yet too afraid to die. This paradox is manifested in the psychopathology of the AN patient who refuses to eat in order to have a meaningful life and to fight against death, as if she is "dying to live". Dally et al.[36] also referred to the close link between eating and death imagery, claiming that the aversion to food among AN patients reflects "a horror of eating", as if to say "you eat, you die", rather than a fear of gaining weight per se. Similarly, Strober[37] argued that AN does not represent a flirtation with death, but actually the struggle to exist within the narrowest parameter. Likewise, Roea et al.[38] contended that the illness itself provides meaning to an otherwise meaningless life, rather than being a threat to it.

Familial interaction was reported as an important etiological factor contributing to the maintenance of AN as early as the mid-19$^{th}$ century[39,40]. The concept of pathogenic/anorexic families continued intermittently, with greater or lesser emphasis, and reached its influential peak in the 1970s[41,42]. Around that time, Hilde Bruch[12] proposed more specific dyadic difficulties as she focused on early mother-daughter interactions in which the mother imposed her own concept of the infant's needs, resulting in a lack of differentiation and sense of separateness. Ultimately, it is this pervasive sense of ineffectiveness that underlies the development of the eating disorder. This point of view is also in line with attachment theory. According to this theory, AN patients do not dare express their interests or needs, but rather feel insecurely attached to others by dependency or avoidance. They lack confidence in the world and the ability to cope with negative emotions.

## DRIVE TO ATTACHMENT IN ANOREXIA NERVOSA

A family environment that is unable to provide a sense of security, availability, and attunement to the child's needs has been identified by attachment theorists as contributing to the pathological dependency or detachment that characterizes a number of mental and eating disorders[43,44]. Adult attachment representations are believed to be internalized working models of infantile drives and associated behaviors. Bowlby postulated a drive that is separate from and more powerful than hunger in its effect on the mother-child relationship: namely, the drive toward attachment[47,48,49,50]. According to Bowlby[45,49], attachment relations endure throughout the lifespan and are fundamental to survival. As children mature, they develop behavioral and mental defensive strategies to promote physical or psychological intimacy[45,49]. These strategies reflect how a parent and a child interact with each other over time in order to maintain a secure base and safety feelings.

One of the main mechanisms of Bowlby's[45,46,47] attachment theory is the view that parental responsiveness, availability, and attunement to an infant's needs can provide the infant with a "secure base" feeling which enables him to regulate his emotional experience and handle distress situations. Since Bowlby developed the attachment theory, there has been some suggestive evidence linking Hilde Bruch's[12] theory of abnormal mother-child interactions and eating disorders with insecure attachment styles later in life. Accordingly, AN patients avoid relying on human beings to fulfill their "secure base" needs, and instead fulfill those needs by resenting and refusing food.

Results of several recent studies support this link of abnormal attachment processes, particularly insecure attachment styles, and eating disorders[44,48,50]. Recent empirical findings[51] show that such insecure feelings may lead to a low sense of mastery and self-worth, hopelessness and helplessness, and ultimately, repulsion by life. As such, AN symptoms may create a false sense of security by providing control over a seemingly meaningless life.

## ATTACHMENT AND DEATH IN ANOREXIA NERVOSA

Little research has been conducted on the connection between attachment style and death. Mikulincer et al.[52] examined the relationship between attachment styles and fear of personal death and found that insecure and ambivalent subjects exhibited a stronger and more overt personal fear of death than did secure and avoidant subjects, and showed a stronger fear of death at a lower level of awareness than did secure subjects. De Jong[53] examined attachment individuation and risk of suicide among adolescents and found that those with a history of suicide rated their parents as emotionally absent in childhood, to a significantly higher degree than did depressed and normal controls. Kaplan and Worth[54] developed a clinical model of suicide in relation to individuation-attachment and Layon et al.[55] further assessed the connection between the threat of separation from a parent and the risk of adolescent suicide attempts.

These results are in contrast with other findings[51] showing that AN patients are insecure and would rather die than gain weight. This discrepancy may be related to the egocentric nature of AN symptoms, which serve as a “false secure base” and as a self-regulation mechanism, thus protecting AN patients from a personal fear of death.

In Bowlby’s terms, secure individuals are less fearful of danger than are insecurely attached individuals because they do not feel that they are helpless or alone. In the same way, the personal fear of death might also be diminished by the sense of permanent support provided by secure attachment bonds. According to Mikulincer et al.[52], it may be that AN patients are not consciously aware of the real threat to their life. Likewise, Bachar et al.[11] suggested that although ED patients do not want to die, they would rather not live in a situation that threatens their ability to control their weight. Their declared preference for dying over gaining weight means that they feel more secure being underweight than being normal or overweight. This fear of gaining weight and the encapsulated AN symptoms may actually serve to protect them from their strong fear of death.

This line of reasoning suggests that attachment style may influence not only the extent of negative feelings experienced in a distressing situation, but also the methods used for coping with the distress. Research examining the relation between the fear of death and attachment style indicates that the cognitive schemata related to previous primary experiences may be generalized to a wide variety of situations, leading individuals to experience fear for the same reason that they are distressed in attachment situations[52].

## CLINICAL IMPLICATIONS

The association between attachment style and the sense of meaning in life may shed further light on family issues and their impact on AN. According to this conceptualization, the question arises as to how clinicians can assist AN patients in feeling secure enough to be attached to life without needing the symptoms, that is, to be attracted to life from a real base of security rather than a false one. Creating an internal sense of security and a “secure base” environment should be the focal point of the therapeutic relationship, rather than focusing the attention and dialog on death and/or preoccupation with food.

In light of the importance of familial factors in the etiology of eating disorders, family therapy may be a critical aspect of treating AN patients and helping them to develop a sense of internal security and autonomy of the self. Family therapy is recommended as a framework for changing family attachment styles through exposure of the beliefs and rules that dictate and influence family functioning, as well as through the introduction of new ways of interacting that promote family stability and a “secure base” environment. Modifications of interpersonal communication and new emotional experiences that occur as a result of transformed interactions with attachment figures may be a powerful way to effect intrapsychic and interpersonal change[56,57].

The desired change may be achieved by giving parents access to round-the-clock acceptance, support, and containment during the first 3 months of treatment (during which time the parents also receive instruction regarding the feeding of their AN daughters). This viewpoint is based on the assumption that if the parents themselves feel a sense of safe communication with the therapist, then they will be able to transmit this feeling to their children. When the parents feel that they are contained and supported, rather than guilt ridden and insecure, they may become more aware of their own strengths and abilities and feel better equipped to take more initiative with their children. In addition, warm and empathic interactions with the therapist may act as a “healing experience”, helping to achieve a more secure attachment style[56,58,59,60].

This approach is in line with the Emotionally Focused Therapy (EFT) model, which addresses attachment issues in the therapeutic process by shifting negative cycles to cycles characterized by affiliation and trust, thereby fostering the creation of a secure attachment bond[61]. Fig. 1 illustrates the therapeutic model that emerged from these theoretical concepts and suggested clinical approach. Hopefully, with this approach, AN patients will start to feel secure enough in life to once again become attached to it.

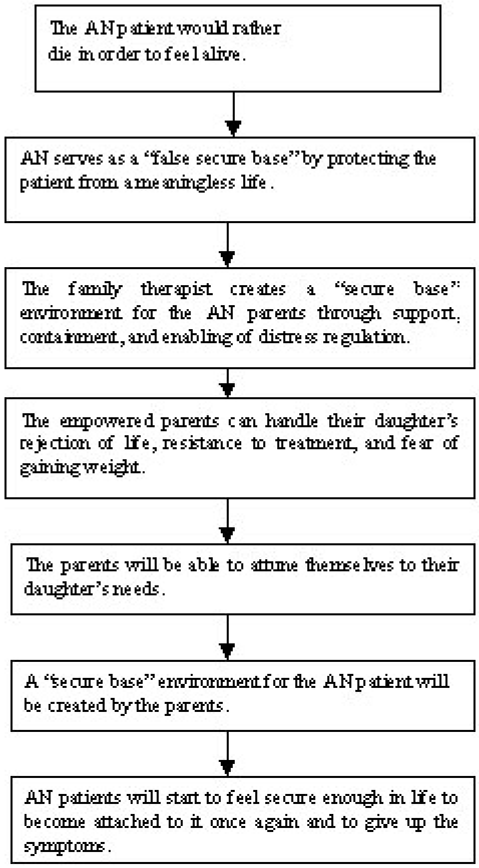

**FIGURE 1.** Therapeutic model: attachment to life through family therapy.

## ACKNOWLEDGMENTS

Portions of this article were based and reprinted, with permission, from our article “Attachment to Life in Anorexia Nervosa” published in *Eating Disorders Review* (2005) **16**, 1. We would like to acknowledge and thank Galia Golan-Sprinzak for her valuable assistance and Sharon Woodrow for her editing and valuable comments.

## BIOSKETCHES

**Yael Latzer**, **DSc,** is the Director of the Eating Disorders Clinic, Division of Psychiatry, Rambam Medical Center, Haifa, Israel, and a Senior Lecturer in the School of Social Work, Faculty of Social Welfare and Health Studies, University of Haifa, Haifa, Israel. E-mail: latzer@zahav.net.il

**Zipora Hochdorf**, **PhD**, is a lecturer in the Faculty of Education, University of Haifa, Haifa and Western Galilee College, Acco, Israel.
